# Text Messages for Depression, Anxiety and Alcohol Abuse Therapy—Are Construction Guidelines Needed?

**DOI:** 10.3390/ijerph192315701

**Published:** 2022-11-25

**Authors:** Teh Faradilla Abdul Rahman, Norshita Mat Nayan

**Affiliations:** 1 Centre of Foundation Studies, Universiti Teknologi MARA, Cawangan Selangor, Kampus Dengkil, Dengkil 43800, Selangor, Malaysia; 2 Institute of IR4.0, Universiti Kebangsaan Malaysia, Bangi 43600, Selangor, Malaysia

**Keywords:** text messages, mental health therapy, construct text message, text message guideline, text message development

## Abstract

Despite the effectiveness of text messaging therapy in improving mental health conditions, limited attention has been paid to how the text messages are constructed. Thus, this study questions whether there is a need to develop a model of text message construction for mental health therapy. In this backdrop, this study reviews how a text message for mental health therapy is constructed, specifically focused on the process and guidelines. This study also aims to identify the research gap regarding the guideline of text message construction for mental health therapy and to identify mental health professionals’ practices in text messaging therapy. In addition, the opinions of mental health professionals on the need to develop a text message construction guideline were also gathered. The findings from the literature review confirmed that there are still limited guidelines explaining the process of constructing text messages for mental health therapy. Meanwhile, results from the online survey found that mental health professionals expressed a high need to explore and develop a model of text message construction for mental health therapy. With this research gap addressed, this study proposes further research into the development of a text message construction model for mental health therapy.

## 1. Introduction

As defined by the World Health Organisation (WHO), “Health is a state of complete physical, mental and social well-being and not merely the absence of disease or infirmity.” People with mental health disorders experience higher disability and mortality rates [1]. The prevalence of major depressive disorder has increased by 27.6% and that of anxiety disorder increased by 25.6% globally due to the Coronavirus Disease 2019 (COVID-19) pandemic [2]. The increase in mental disorder cases is due to life burdens during the pandemic such as financial crises, lost jobs, movement control, family members becoming infected with diseases or domestic violence experienced during the lockdown [2,3]. People with mental health disorders may have problems managing themselves, live unhygienic lives, fail to hold work responsibilities or have no access to normal education. In addition, poor mental health conditions negatively impact the individual in terms of social life, such as denial of the right to marry or have a family, as well as the tendency to be exposed to sexual and physical abuse or be excluded by society [1].

In recent years, mental health has increasingly become a core area of interest especially related to the importance of its care. Many aspects of mental health care have increasingly become the focus of researchers since the COVID-19 pandemic [4,5,6,7]. Traditionally, mental health problems are treated through in-person counselling or medications at mental health clinics. Due to factors such as the interruption of mental health services, transport barriers to visiting physical clinics or negative stigma around receiving in-person treatment have led to the use of digital mental health therapy. Some digital platforms such as smartphone apps, text messaging and web-based solutions have been used by previous studies to treat different kinds of mental health problems [8,9,10,11]. Among these digital platforms, text messaging through basic cell phone service is the most cost-effective and promising approach to reach patients easily.

Every virtual platform has its own functions, interactions, uniqueness and content. SMS, for example, has been widely used to support changes in the attitudes of individuals suffering from depression and anxiety [5,12,13]. Despite the effectiveness of text messaging in treating mental health problems, limited attention has been paid to how these text messages were constructed. The guidelines to build text messages for the purpose of delivering mental health therapy are extremely limited [14].

Studies show that employees with mental health problems are seven times more likely to be unproductive compared to employees with better mental health [15]. There are organisations that have invested in mental health programs for their staff. These organisations hope the program can improve employees’ mental health conditions so that they can perform tasks well without affecting productivity. However, the absence of a guide for the construction of text messages used by mental health professionals may cause deterioration in the effectiveness of treatment. As a result, the mental health of employees is still at a poor level, which increases the cost of organising mental health programs in the future and causes losses when employees are absent from work as a result of poor mental health.

Topics for online text message therapy, especially for treating mental health problems, need to be understood so that they are effective not only in terms of improving a person’s mental health but also to ensure that the therapy delivered through an application does not cause participants to withdraw from participating in therapy in the future. With additional support available, it may help the participant remain in online mental health treatments. The research problem above leads to the question of whether it is necessary to develop a model of text messages construction for mental health therapy. In addition, what topics are suitable for the therapy of a mental health problem?

Therefore, this study reviews the text message construction approaches used in previous studies and identifies mental health professionals’ practices in text messaging therapy to provide evidence for the need to develop a text message construction model for depression, anxiety and alcohol abuse therapy. If such a model is found to be a necessity, further research into appropriate text message construction for depression, anxiety and alcohol abuse therapy will be needed.

## 2. Literature Review

People with mental health problems can be helped through treatments. Mental health treatments include the nonmedical approach of psychotherapy, such as reality therapy, gestalt therapy, cognitive behavioural therapy (CBT), narrative therapy and solution-focused brief therapy. Other than that, the treatment also includes mental health counselling and psychoeducation [16]. These treatments are delivered to the client not only through a face-to-face approach, but also via the digital environment. For example, text messages for mental health have been used to deliver CBT therapy to individuals with alcohol use problems [17], while mobile apps that contain messages focusing on behavioural change techniques have been used to reduce alcohol consumption among the veteran population [18].

The design of text messaging therapy for mental health varies based on different types of psychotherapy approaches, session lengths, telecommunication media, frequencies of text messages sent, one-way or two-way communications and targeted outcomes. Lattie et al. [19] summarised a list of domain considerations provided with recommendations for designing a digital text-based mental health coaching protocol. The recommendations include the an intervention goal, expectations, message frequency, coach characteristics and training, coaching model, progress monitoring, message length, message content priority, instruction and questions as well as other types of communication media to support a specific goal [19].

In a study related to the delivery of support in suicide risk management [20], the initial development of the text message began with the determination of relevant theories: psychological theories and principles of motivational interviews. The selected theories also met the objectives of therapy that have been determined in advance, which was to encourage the use of individual coping strategies identified as part of safety planning, access to various types of support and to the use of additional coping tools and resources. Next, text messages were built focusing on self-efficacy to control and avoid suicide, motivation to maintain safety, customisation of text messaging with personal safety plan strategies, additional coping tips and strategies, references to crisis helplines, encouragement to use a personal safety plan and reinforcement of social interaction.

Toyama et al. [21] designed text messaging therapy with the goal to encourage patients to seek mental health care services. In the text message development phase, a set of questionnaires containing 26 open- and closed-form questions was constructed with the help of a psychiatrist. The questionnaire asked about participants’ opinions on SMS components, participants’ readiness to receive SMS therapy, the choice of the number of SMS messages to be received, the frequency of text messages and the appropriateness of delivery times [21]. In addition, interviews with participants were also conducted using open-ended questionnaires. The literature review was helpful in identifying how different techniques and components were considered during text message construction. However, there is still a need to further explore the steps and processes that were implemented by previous studies and current mental health practitioners in constructing text messages for mental health therapy.

## 3. Materials and Methods

**Figure 1:**
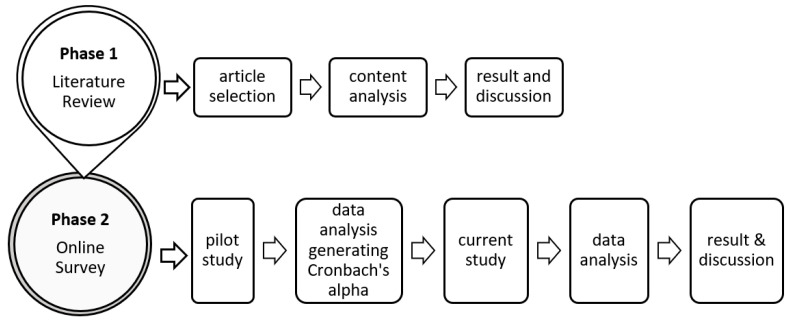
Methodogy system flow.

**Figure 2:**
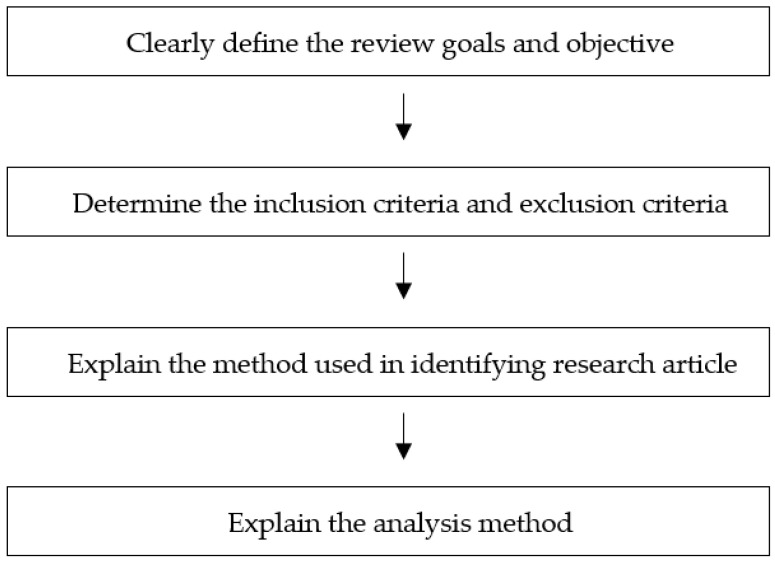
Stages of Literature Review [22].

This study consists of two phases, as shown in Figure 1. In the first phase, a literature review was conducted according to the guidelines introduced by Nightingale [22]. Figure 2 shows the stages involved in the implementation of the literature review [22]. The first stage in conducting a systematic survey was to develop a protocol that clearly defines the goals and objectives of the survey.

The aim of this literature review was to identify models or approaches of text message construction for any mental health therapy used in previous studies. The purpose is to understand what has been implemented by other studies in constructing a text message and to identify the research gap that can be filled in future studies. To select research articles to be included in the review, the inclusion criteria and exclusion criteria were determined. In this study, articles must (1) be written in English, (2) study text messaging intervention, (3) be targeted at any mental health problem, (4) explain the effectiveness of the pre- and post-text messaging intervention and (5) mention the type of intervention. An article is excluded if (1) it does not fulfil the inclusion criteria, (2) it is a type of intervention protocol paper or (3) it is a review paper.

Next, data sources and search strategies were implemented based on the guidelines introduced by scholars [22,23]. A total of four databases, namely, Scopus, ACM Digital Library, IEEE Xplore and Web of Science, were used for article searching. During the search process, keywords such as ‘mhealth’, ‘Whatsapp’, ‘SMS’, ‘chat’ and ‘mobile text messaging’ were entered into the search engines targeting the types of text messaging telecommunication platforms. Apart from that, keywords related to the type of mental illness were also used such as ‘depression’ and ‘anxiety’. Each keyword was combined using the logical operators ‘AND’ or ‘OR’ to obtain more relevant results. In the final stage of the search strategy, abstract reading was carried out to extract articles that were not relevant to the context of the study. In addition, the title and abstract filtering process was performed on each article repeatedly to ensure that no similar studies were included in the list. Next, further reading was conducted on the selected articles by focusing on the methods and findings of the study presented in each article. Readings were carried out from page to page to identify the gaps that can be filled and their importance. Finally, research articles that did not meet the inclusion criteria were removed from the reading list.

Analysis was conducted on the selected articles using the content analysis method [24]. Firstly, all the selected articles were grouped in a folder and given a unique identification number. Secondly, preliminary readings were performed on each selected article to obtain general information and an overview of the background of the study that was conducted by previous researchers. Thirdly, a coding method was used in which keywords, sentence fragments and paragraphs were broken down into several categories. These categories are models, theories, resources and content construction methods. Then, some of the categories were grouped as appropriate by the first author. Next, the categories found in the content analysis were reviewed by the second author of this study to determine the accuracy of the qualitative findings. Through review and discussion, the terms for the categories presented were improved. After that, a discussion was held with another researcher who has an interest in the same field of this study to increase the accuracy of the categories. He reviewed the final report that had been written, then asked questions about some aspects of the background of the study that were unclear. As a result, he agreed and confirmed the accuracy of the categories extracted from the content analysis. In the next step, reading of the articles was repeated to analyse the aspects that have the potential to be considered as themes. In the fifth step, the results of the review are presented in the form of descriptions and tables. The final step in the content analysis involved the process of interpreting the information obtained from the findings. In this step, the researcher summarises the main ideas that can be learned from the review.

In phase two, a survey was conducted to identify mental health professionals’ practices of delivering mental health problem therapy through text messaging and to gather their opinions on the need for the development of a text message construction model. The findings of this stage are important to reinforce the need to continue the development of text message construction models for depression, anxiety and alcohol abuse therapy. Respondents at this stage were mental health practitioners: nurses, psychiatrists, counsellors and doctors. A total of 30 respondents were involved in the pilot study, whereas 82 respondents were involved in the current study. For this study, non-random convenience sampling was used to reach respondents consisting of mental health practitioners (population).

Invitations to participate in the study were sent via email and the WhatsApp text messaging platform. A self-administered questionnaire was conducted through the Google Form platform consisting of three sections: (A) consent declaration form, (B) occupational field background and (C) opinion and practices. The items in section C were developed and modified from previous studies [25,26]. Seven items in the section require the respondent to give the answer “Yes” or “No”, while one item (item no. 5) requires an answer regarding the source of text message construction method if the respondent answers “Yes”.

Data collected from the pilot study were used to test the reliability of the study items by generating Cronbach’s alpha values using the Statistical Package for Social Science (SPSS) 24 software. Data from the current study were analysed using descriptive statistics including frequency and percentage by using Microsoft Excel 2016 software. The data for item number 5 were analysed using content analysis method if any respondents included text message construction resources.

## 4. Results

### 4.1. Phase 1: Literature Review

**Figure 3:**
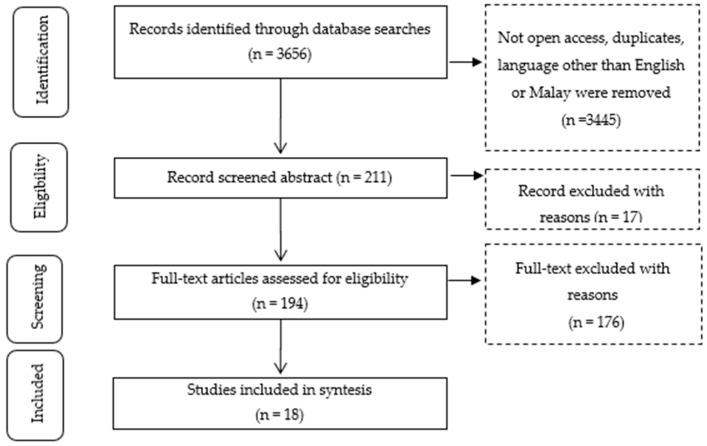
Article Selection Flow [27].

The search was carried out on articles published from 2013 to 2020. The seven-year interval was chosen to obtain better and more mature research findings. An initial literature search found a total of 3656 articles in the database records based on keywords entered during the search process. The article selection flow is shown in Figure 3.

After the screening process, a total of 18 articles were selected for review (Table 1).

The process of coding the 18 articles resulted in two categories, labelled with the terms ‘Basis of Study Methods’ and ‘Text Message’, respectively. After initial reading and analysis through coding of the 18 articles, re-reading was performed in the fourth step to analyse potential themes under each category. This resulted in two themes under the category of ‘Basis of the Study Methods’, namely, ‘Psychological Models’ and ‘Theory of Psychology’. The ‘Text Message’ category was combined into one theme, namely, ‘Text Message Construction Method’. Based on the findings shown in Table 2, only two out of all the articles reviewed stated the application of psychological models and theories as the basis of the study. Sixteen of the previous studies examined did not state the use of any psychological model, whereas ten studies in the sample articles stated the use of a psychological theory, with six of them applying cognitive and behavioural theory and the other four studies applying behavioural theory. The remaining studies (n = 8) did not state the use of psychological models and theories.

Text messages were used as interventions in 9 of the 18 studies and were constructed through various approaches. These approaches were: text messages were constructed using human resources by holding discussion sessions with a group of mental health experts and patients (n = 3), discussions with patients only (n = 2) and discussions with experts only (n = 1). A more organised and structured text message construction method was used by two studies with reference to a procedure [43]. Meanwhile, one study [36] used a health guide manual as a reference in the construction of text messages; however the study did not explicitly state the processes involved. On the other hand, another half of the total number of studies examined did not specify the text message construction methods.

Bock et al. [35] developed a text messaging intervention to treat alcohol addiction among community college students. The study involved mental health practitioners and patients to construct and refine the text messages. To begin with, a set of sample text messages constructed by the research team was given to the students as users. The sample text messages were divided into three categories: safer drinking strategies, myths and facts about alcohol consumption and links to related online content. Next, the users were asked to provide feedback on the content, tone and format of the text message. The users were also asked to describe the type of text message that would be appropriate to send to a friend with the goal of encouraging that friend to practice safe alcohol consumption. The results of user feedback suggested that the text messages that should be built are factual, increase the motivation to consume alcohol safely and are constructed according to the background of the target group. In the next step, eight students who drink alcohol and use text messaging services were appointed to construct an alcohol addiction text message. The text message was built around the topic of the results from the previous stage and an appendix of facts on safe consumption of alcohol prepared by experts. It was also composed in a more relaxed tone, as if written by a peer. Next, completed text messages were reviewed by the researchers of the study independently. The text messages that were preferred by at least three raters were selected for use in the actual intervention.

Kodama et al. [33] conducted a discussion with mental health patients to understand their needs before constructing text messages. The text messages were constructed by taking into account psychiatric outpatients’ background of having suicidal ideation as assessed by a psychiatrist. In addition, aspects of the topic, the length of the text messages, the time the text messages were sent, the frequency of the text messages and individual customisation were things that were considered in the construction of the text message. In this study, there was no statement about the model or theory relied upon when constructing text messages. A total of 52 text messages were created by psychiatrists targeting stress management, maintaining good mental well-being, encouraging medication adherence, methods of improving sleep, the importance of talking to someone about problems and information about services. Then, the constructed text messages were reviewed by two psychiatric outpatients. However, it is not clear whether feedback was collected or whether there were improvements made as a next step.

The method of text message construction used by Agyapong et al. [28] relates to the cognitive and behavioural theories and involves discussions with mental health practitioners and patients. The study did not clearly describe the steps taken when constructing text messages before they were used in the intervention. However, the identification of mental health problems and the characteristics of targeted participants was performed. Text messaging is a form of support with the aim to improve emotions and overall health as well as reduce the number of visits to health services. The principles of cognitive behavioural therapy were used as a backup in the construction of text messages. The messages were written by a cognitive behavioural therapist in collaboration with the patient and were pre-programmed into an online software that delivered messages at 10 am and 7 pm every day. Two different text messages were sent each day, with no repetition of the same message over a 90-day period. The same text message was sent to all patients without considering individual customisation aspects such as name, age, or gender. This reading found no text message review activity in its writing.

Renfrew et al. [42] stated the use of the psychological model and theory as the basis of the text messaging intervention. However, there was no detailed explanation on the construction process of text messages sent to the intervention participants. Constructed text messages focused on the process of completing target behaviours, such as reading text content and engaging in experiential learning, as opposed to outcomes that may be harmful and beyond the individual’s control. Each text message sent contained the participant’s name, a predetermined message to encourage engagement, and was signed by a member of the research team. In addition, text messaging is based on the theory of planned behaviour. In his study, no information about the groups of individuals involved in the construction or review of text messages was found.

Xu et al. [30] built text messages based on the health belief model, behavioural theory, and discussions with mental health practitioners. The researchers selected four individual components to be used in their intervention from the empirical literature on task sharing, medication adherence, and mHealth to improve patient adherence to medications. The four components are lay health supporters, e-platform, award and integration. Lay health supporters are family or community members, whereas the e-platform is the existing commercial telemarketing system used to send text messages. The award is a simple appreciation message sent as a token gift when a user actively participates in the intervention. The last component makes use of text messages as a communication tool that integrates the efforts of lay health supporters into the existing health system. In the initial stage, the patient’s background and objectives were identified before the text message was sent. Then, the program was set to send two text messages per day to patients and public health aides. The first text message, sent at 9 am, contained educational information about schizophrenia, while the second text message was sent at 7 pm as a reminder to take medication. All messages were expressed in a loving, polite and personal style of language and tone because those characteristics were preferred by the patient. A group of master’s and doctoral students in public health and medicine were tasked with producing text messages by adapting content from evidence-based sources. From that, a total of 237 educational text messages were successfully constructed. Next, a senior psychiatrist reviewed and approved the messages for use. The article does not clearly discuss the relevance of the psychological theory as a basis for the construction of text messages. In addition, there was no detailed explanation of the review conducted by experts including from the aspect of review repetitions, text message improvement measures, spelling, meaning and appropriateness of the topic with the patient’s condition. The review stage also did not involve obtaining opinions and feedback from the treated patients.

Aguilera et al. [36] conducted a study using mood-monitoring and treatment-related text messages to test whether such messages could increase engagement and improve clinical outcomes in CBT treatment for depression. The text messages’ content was developed based on the Building Recovery by Improving Goals, Habits, and Thoughts (BRIGHT) manual and cognitive behavioural principles that generally focused on cognitive, self-monitoring, behavioural activation, interpersonal interactions and healthy behaviours that affect emotions. The constructed text messages were then arranged according to the weekly module learning. It was not stated whether the completed text message went through a review process before being used in the intervention. However, improvements to text messaging were made while the intervention was ongoing based on ongoing feedback from patients and knowledge gained from the recent mHealth field research. It aimed to improve the usability of text messaging in treating depression. Overall, the text message construction method used by this researcher was seen to be very organised and was guided by principles in the field of psychology. However, it lacked clarity in terms of word count, language style and tone, review stage and text message length.

In another study, Agyapong et al. [39] again implemented cognitive and behavioural theories and discussions with mental health practitioners and patients to construct text messages for alcohol addiction therapy. Text messages were built in support of and related to alcohol abuse. In addition, text messages were formulated based on the principles of addiction treatment to target the prevention of alcohol use. The messages were written by counsellors in the field of addiction in collaboration with patients. Constructed text messages were then reviewed and modified to a maximum of 160 characters by a group of experts consisting of psychiatrists, addiction counsellors and social workers. Next, the text messages were pre-programmed into the online software by sorting each text message. As in the author’s previous study [28], this program also delivered text messages to patients automatically twice a day for a period of three months. Every day, the patient receives a different text message. Text messages were sent at 10 am and 7 pm based on the good acceptance of those times by patients from previous studies.

Shingleton et al. [32] conducted a study on the effectiveness of motivational text messages on individuals with eating disorders. The study implemented cognitive and behavioural theories and conducted discussions with patients. Before the text messages were constructed, patients’ information was collected, including demographics, height and weight, eating disorder problems and other psychological symptoms such as depression, suicidal ideation, psychosis and substance use. Then, patients were asked to complete a questionnaire and attend a motivational interviewing session. The interview session was conducted face-to-face and lasted approximately 1 h to collect information about the patient’s goal in treating their eating disorder problem. Next, 60 customised text messages were created for each participant. Motivational text messages were personalised to reflect the domains of content discussed during the interview session by patients who were negatively affected by their eating disorder problems. Among the domains mentioned were relationships, work, school and physical health. Additionally, message content was designed as an adjunct to CBT for eating disorder problems that focused on normal eating and reducing the symptoms. To align with the principles of CBT, the text messages were designed to target restrictive eating behaviours and consequences. Overall, the method used was very good as it analyses the needs of the targeted individual before a text message was constructed. In addition, the content of the text message was constructed based on the patient’s stated needs, making it more meaningful and has the potential to reduce symptoms. A limitation of the method is that it does not explain the review aspect before the text message is used in the actual intervention.

### 4.2. Online Survey

The purposes of the online survey distributed to the mental health professionals are to identify mental health professionals’ practices in text messaging therapy and to provide evidence for the need to develop a text message construction model for depression, anxiety and alcohol abuse therapy. The acceptable Cronbach’s alpha value is over 0.70 [44]. Based on the pilot study, the Cronbach’s alpha value for the set of questionnaires containing eight items was 0.725. Therefore, the set of questionnaires used in this study was considered good and suitable to be used for the current study. Based on Table 3, 64 out of 82 respondents stated that they have delivered mental health therapy through text messaging before. Only 6% of the respondents did not think text messaging could support patients with mild levels of depression, anxiety and alcohol abuse. In addition, the majority of the respondents agreed that there is a need to conduct therapy not just during the COVID-19 pandemic (84%) but also in any other situations (77%). On the other hand, only eight of the total respondents stated that there is a text message construction guide that focused on the therapy of depression, anxiety and alcohol abuse, but only four of them filled the resource information. Of the four responses, only one person provided the resource link. The remaining respondents did not provide answers related to guide sources. Other than that, 98% of the respondents agreed that text messaging can be constructed with the right steps to ensure that appropriate therapy can be delivered to individuals with specific mental health problems. Moreover, 91% of the respondents agreed that the process of constructing appropriate text messages for the therapy of depression, anxiety and alcohol abuse needs to be based on diverse topic elements, words and sentence types. Lastly, 96% of respondents stated that there is a need to develop a text message construction model for the therapy of depression, anxiety and alcohol abuse.

## 5. Discussion

Overall, most articles do not describe in detail the method of constructing text messages sent to patients during the intervention. Through reading, it can be understood that past researchers have sought consultation from experts in the field of mental health or have referred to scientific materials for the purpose of building text messages. This finding is consistent with a previous literature review [14] on the involvement of key informants through interviews or focus group discussions, where engagement with the participants who will receive the text messages and mental health professionals could help refine message content. This has the potential to increase improvement in the targeted behaviour change. However, based on the review, previous studies failed to explain the methodology of text messages construction for the use of mental health therapy. This finding is the same as mentioned by other reviews, which says that there is minimal guidance on messaging content development [14]. A past review of the reviews [45] also found and focused on the effectiveness, feasibility, acceptability and economic evaluation in different contexts of mental health, but not on text messages’ construction method. Therefore, it is still unclear how the construction process is implemented and how models and theories are taken into account in therapy for a targeted mental health problem. These findings prove that there is ambiguity in how a text message is constructed before it is sent to the patient and whether it is built to tailor to the type of the mental health problems faced by the individual concerned. Based on the findings, these are the gaps that need to be filled in the text messaging therapy field study. As a summary of the content analysis of the 18 articles, there are still limited studies focusing on text message construction models for the therapy of mental health problems. The findings also provide an initial knowledge and background of the needs and necessity to develop a model of text message construction for depression, anxiety and alcohol abuse therapy. This text message construction model is considered very necessary because it can be used as a guide by mental health practitioners who wish to implement text message-based therapy.

The majority of the respondents practice text messaging as a medium of mental health therapy. Although some respondents did not practice text messaging therapy, they still believe that this type of therapy method could provide support to patients with depression, anxiety and alcohol abuse. Surprisingly, despite the findings showing that most respondents practice text messaging therapy, the majority did not know if there is a source of text message construction guide that can be referred to. However, the respondents were aware of the need to use the right steps and diverse topic elements, words and sentence types while constructing text messages for psychotherapy. Only four respondents filled the resource information on text messages’ construction guide. After analysing the resources provided, it was found that there is still less attention paid to the text message construction processes and stages involved. Thus, it is not surprising that almost all of the respondents agreed that it is necessary to develop a text message construction model.

There are various factors that need to be considered before building a text message. According to Whealin et al. [46], developers should take into account variations in participant demographic factors and their needs. This is because the goal of health behaviour change will be chosen based on a balance of health preferences, individual characteristics and the level of readiness to change [47,48]. For example, the individual may be a pregnant smoker and the proposed behaviour change goal is to stop smoking among those who are ready. In addition, developers must ensure the compatibility and suitability of the text messages with the goal to be achieved from the intervention [49]. The factors of readability level, cultural sensitivity [46,47], clarity and grammar also need to be considered [43]. Literacy demands are important to improve understanding [49] as well as to avoid the patient from becoming bored or losing focus. The use of a decision tree approach that determines when and what type of message should be sent can help tailor the text messages to individual treatment [50]. Although most past researchers did not explain in detail the relationship between the frequency and time of sending text messages with the construction, it can be seen that both are important aspects of the text messages’ construction because they help researchers plan content that will not burden the patient and at the same time increase treatment effectiveness and patient engagement [51].

The results in this phase are captivating on many levels. First, this study expressed mental health professionals’ belief that text messaging therapy may support the patients in many ways despite some of them not practicing online therapy. It is understandable that some mental health professionals contend that text messaging therapy becomes a need in many situations. As stated by previous literature, this is due to some limitations faced by the therapists or by the patients such as lack of technology accessibility, low technology, literacy and difficulties in reading and understanding text messages [52,53,54]. Second, the study also indicates that the choice of topic, word and sentences are important to be tailored to the patients’ background. The positive effects or gained-frame text messages sent to the patients are more effective in enhancing mental health [55]. Participants in a study also mentioned that they prefer to receive positive and motivational message content [52]. Third, the finding has implications for the need to develop a model for text message construction. Best practices do exist for developing SMS message content for behaviour change, but further research is still needed to refine the processes involved in the text message content development [14]. Future research on the components and elements of text message construction model involving therapy content, psychological model and theories could shed more light on these issues.

## 6. Conclusions

This study delved into the explanation of noteworthy applications and methods of text message construction, focusing on understanding the processes of previous studies. An approach that has been used by most of the past researchers is entirely dependent on the expertise and knowledge of mental health experts as well as the level and nature of the patients’ involvement. Very little research has described the text message construction process clearly and in detail. This is also supported by mental health professionals’ opinions regarding the need to develop a text message construction model for depression, anxiety and alcohol abuse therapy. This research also provided insight into mental health professionals’ practices and opinions in the context of text messaging therapy. The findings of this research have a significant impact for the provision of digital mental health because text messaging in depression, anxiety and alcohol abuse therapy is now widely taking place on various communication platforms. To conclude, this study strongly emphasises the need to develop a text message construction model for depression, anxiety and alcohol abuse therapy. Mental health practitioners can use the model as a guideline to improve text message content development.

## Figures and Tables

**Table 1:** References of Articles Selected for Literature Review.

ID	Author	ID	Author
S1	Agyapong et al. [28]	S10	Menezes et al. [29]
S2	Xu et al. [30]	S11	García et al. [31]
S3	Arean et al. [8]	S12	Shingleton et al. [32]
S4	Kodama et al. [33]	S13	Anstiss & Davies [34]
S5	Almeida et al. [12]	S14	Bock et al. [35]
S6	Aguilera et al. [36]	S15	Zhang et al. [37]
S7	Christensen et al. [38]	S16	Agyapong et al. [39]
S8	Islam et al. [13]	S17	Wolf et al. [40]
S9	Kraft et al. [41]	S18	Renfrew et al. [42]

**Table 2:** Theory, Model and Text Message Construction Method as Reported in Selected Articles.

	Basis of the Study Method						Text Message					
Article ID	Psychological Model			Theory of Psychology			Text Messages Construction Method					
	Health Belief Model	Supportive Accountability Model	None	Cognitive and Behavioural	Behavioural	None	Discussion with Mental Health Practitioners and Patients	Discussion with Patients	Discussion with Mental Health Practitioners	Structured	Health Manual	None
S1			√	√			√					
S2	√				√				√			
S3			√			√						√
S4			√			√	√					
S5			√			√				√		
S6			√	√							√	
S7			√	√								√
S8			√		√					√		
S9			√			√						√
S10			√		√							√
S11			√			√						√
S12			√	√				√				
S13			√	√								√
S14			√			√		√				
S15			√			√						√
S16			√	√			√					
S17			√			√						√
S18		√			√							√
sum	1	1	16	6	4	8	3	2	1	2	1	9

**Table 3:** Items on Online Survey.

Item	Frequency, Percentage (n, %)	
	Yes	No
1. Do you deliver mental health therapy through text messaging as an alternative to face-to-face treatment?	64 (78%)	18 (22%)
2. In your opinion, can text messaging to some extent provide support to individuals experiencing mild levels of depression, anxiety and alcohol abuse?	77 (94%)	5 (6%)
3. In your opinion, is there a need to conduct therapy of depression, anxiety and alcohol abuse (mild levels) via text messaging during the COVID-19 pandemic?	69 (84%)	13 (16%)
4. In your opinion, is there a need to conduct therapy of depression, anxiety and alcohol abuse (mild levels) via text message in any situation? (Example; during a pandemic or not, clinical services are difficult or easily accessible, the level of readiness of the individual to seek treatment, etc.)	63 (77%)	19 (23%)
5. To the best of your knowledge, is there a text message construction guide that focuses on the therapy of depression, anxiety and alcohol abuse?	8 (10%)	74 (90%)
6. In your opinion, should text messaging be constructed with the right steps to ensure that appropriate therapy can be delivered to individuals suffering from depression, anxiety and alcohol abuse?	80 (98%)	2 (2%)
7. In your opinion, should the process of constructing appropriate text messages for the therapy of depression, anxiety and alcohol abuse need to be based on diverse topic elements, words and sentence types?	75 (91%)	7 (9%)
8. Is it necessary to develop a text message construction model for the therapy of depression, anxiety and alcohol abuse?	79 (96%)	3 (4%)

## Data Availability

Not applicable.
